# Consultants or Trainees: Whose patient's do better following surgical coronary revascularisation?

**DOI:** 10.1186/1749-8090-10-S1-A348

**Published:** 2015-12-16

**Authors:** Damian Balmforth, Daniel Jones, Sean Gallagher, Andrew Wragg, Alex Shipolini, Kit Wong, Rakesh Uppal

**Affiliations:** 1 Barts Heart Centre, St Bartholomew's Hospital, London, EC1A7BE, UK

## Background/Introduction

It is a commonly held belief that patients undergoing coronary artery bypass grafting (CABG) have better outcomes when their surgery is performed by a consultant rather than a surgical trainee. However, there are few studies that analyse the relationship between operator grade and clinical outcomes.

## Aims/Objectives

To investigate the effect of operator grade on the outcomes of CABG in the setting of a major tertiary centre with a nationally accredited cardiac surgical training programme.

## Method

A retrospective observational cohort study was performed on prospectively collected data for all patients undergoing CABG between January 2003 and July 2011.

## Results

A total of 6689 patients underwent CABG of which trainees performed 1968 (29.4%). The proportion of procedures performed by trainees declined over time from 30.2% in 2003 to 26% in 2010. Consultants tended to perform more high risk operations with their patients being more likely to have high Euroscores, be urgent cases, and have cardiogenic shock. In the unadjusted Cox analysis, consultant operator grade was associated with an increase in 5 year mortality [HR: 1.26 (95% CI: 1.07-1.47)]. However, this association did not persist following multiple adjustment for co-morbidities [HR: 1.02 (95% CI: 0.87-1.20)]. In a propensity score analysis that stratified patients by risk, whilst consultants were found to have performed a greater proportion of high risk cases, there was no significant difference seen in 5 year mortality between consultants and trainees across the range (RR 1.04 (95% CI: 0.86-1.24)].

## Discussion/Conclusion

There is no difference in clinical outcomes between trainees and consultants where CABG is performed in a centre with a dedicated training programme under direct consultant supervision.

